# Draft Genome Sequence of Urease-Producing *Sporosarcina pasteurii* with Potential Application in Biocement Production

**DOI:** 10.1128/genomeA.01256-13

**Published:** 2014-01-30

**Authors:** Pradeep Kumar Tiwari, Kandarp Joshi, Rakhshinda Rehman, Vivek Bhardwaj, K. V. Shamsudheen, Sridhar Sivasubbu, Vinod Scaria

**Affiliations:** a Genomics and Molecular Medicine Unit, CSIR Institute of Genomics and Integrative Biology, Mathura Road, Delhi, India; b CSIR Open Source Drug Discovery Unit, Anusandhan Bhavan, New Delhi, India; c GN Ramachandran Knowledge Center for Genome Informatics, CSIR Institute of Genomics and Integrative Biology, Delhi, India; d Academy of Scientific and Innovative Research (AcSIR), Anusandhan Bhavan, New Delhi, India

## Abstract

We describe here the draft genome sequence of *Sporosarcina pasteurii*, a urease-producing bacterium with potential applications in biocement production.

## GENOME ANNOUNCEMENT

The use of microbes in biomineralization has been an active area in research worldwide (1, 2). One of the major microorganisms studied for its biomineralization capacity has been the urease-producing species *Sporosarcina pasteurii*, which has the potential to precipitate calcium carbonate and thus aid in the production of biocement (3, 4). The organism produces a significantly high amount of urease without the requirement for additional methods of concentration (3, 4). *S. pasteurii* releases carbonate ions, which in a calcium-rich environment and under adequate conditions can precipitate hard calcium carbonate or calcite (5). A recent crystallographic study on the citrate-bound urease enzyme complex shed light on the mechanisms of action of this enzyme (6). The organism has an additional advantage in that it tolerates a highly alkaline environment, which is required for biomineralization (7). The microorganism has also gained interest and applications in a wide variety of areas, including the restoration of structures and mining (8), and it may have biomedical applications. The microorganism also provides an ecofriendly alternative to traditional cement production systems.

The strain was procured from the National Collection of Industrial Microorganisms (NCIM) maintained at the CSIR National Chemical Laboratory, Pune, India (NCIM ID, 2477). DNA was isolated from the culture, which was revived from the freeze-dried samples. The raw sequence data were generated after library preparation on an Illumina HiSeq 2500 platform as per the manufacturer’s protocols. A total of >11.5 million single-end reads of 101 bases were generated. The draft genome was assembled *de novo* using the CLC Genomics Workbench 6.0.5. The assembly resulted in 38 contigs with an N_50_ value of 232,940 bp and a total assembly of 4,040,808 bp. Further, automated gene prediction on the draft genomes was performed using RAST server (9). The analysis revealed 4,259 genes, including 46 RNA genes.

### Nucleotide sequence accession numbers.

This whole-genome shotgun project has been deposited at DDBJ/EMBL/GenBank under the accession no. AYOX00000000. The version described in this paper is version AYOX00000000.1.
